# Descriptive Analysis of Components of Emergency Medicine Residency Program Websites

**DOI:** 10.5811/westjem.2021.4.50135

**Published:** 2021-07-15

**Authors:** Jordan R. Pollock, Jeffery A. Weyand, Amy B. Reyes, Shiva Senemar, Aunika L. Swenson, Rachel A. Lindor, James L. Homme

**Affiliations:** * Mayo Clinic Alix School of Medicine, Scottsdale, Arizona; † Washington State University Elson S. Floyd College of Medicine, Spokane, Washington; ‡ Arizona State University, Tempe, Arizona; § Stanford University, Department of Emergency Medicine, Palo Alto, California; ¶ Mayo Clinic, Department of Emergency Medicine, Phoenix, Arizona; || Mayo Clinic, Department of Emergency Medicine, Rochester, Minnesota

## Abstract

**Introduction:**

Most emergency medicine (EM) applicants use the internet as a source of information when evaluating residency programs. Previous studies have analyzed the components of residency program websites; however, there is a paucity of information regarding EM program websites. The purpose of our study was to analyze information on EM residency program websites.

**Methods:**

In April–May 2020, we evaluated 249 United States EM residency program websites for presence or absence of 38 items relevant to EM applicants. Descriptive statistics were performed, including means and standard deviations.

**Results:**

Of the 249 EM websites evaluated, the websites contained a mean of 20 of 38 items (53%). Only 16 programs (6%) contained at least three-quarters of the items of interest, and no programs contained all 38 items. The general categories with the least amount of items were social media use (9%), research (46%), and lifestyle (49%), compared to the other general categories such as application process (58%), resident information (63%), general program information (67%), and facility information (69%). The items provided by programs most often included program description (98%), blocks and rotations (91%), and faculty listing (88%). The items provided least often included housing/neighborhood information (17%) and social media links (19%).

**Conclusion:**

Our comprehensive review of EM residency websites in the US revealed the absence of many variables on most programs’ websites. Use of this information to enhance accessibility of desired information stands to benefit both applicants and programs in the increasingly competitive specialty of EM.

## INTRODUCTION

Emergency medicine (EM) is a popular specialty among medical students, evidenced by the growing number of EM residency applicants over the last 10 years, with 2903 applicants in 2011 and 3640 in 2020.^1^ Many applicants depend on the internet as a primary source of information when researching different residency programs.^2^ This has been particularly true for the 2020–2021 residency cycle due to COVID-19-related restrictions on travel and in-person activities. Even prior to this change, information available on the internet was often a determining factor in prospective applicants’ decisions to apply for rotations or residency interviews. A survey of EM applicants found that 78% claimed information provided in the residency program website influenced their decision to apply to a particular program.^3^ In addition, 41% of applicants decided not to apply to at least one program solely based on the information available on the residency program website.^3^ Accordingly, website development, content, and accessibility are increasingly important for residency programs.

To assist medical students in navigating the staggering number of residencies across the United States, databases such as the Fellowship and Residency Electronic Interactive Database (FREIDA) have been designed to allow convenient access to program websites and information on residencies and fellowships.^4^ However, little is known about the quality of information available through these websites in the field of EM. Prior studies have evaluated residency websites for other specialties,^5^–^9^ resulting in various recommendations for areas of improvement among their respective program websites to both help applicants and increase recruitment.^6^,^9^–^11^ The main purpose of our study was to provide an in-depth analysis of EM residency website content for prospective EM applicants. To our knowledge, this study is the first to evaluate these variables in the specialty of EM.

## METHODS

This study was exempt from institutional review bgoard approval because it involves publicly available information. Our methods were adapted from a previous study analyzing otolaryngology residency websites.^6^ We obtained a list of 256 EM programs from FREIDA in April 2020. When a link to a program was not available on FREIDA, we performed a Google search to find the program website. Residency programs without a functional residency website or a website that could not be found were excluded. When two websites were available for the same program, we used information from both the institutional and the non-institutional program website. We did not include Facebook, Instagram, or other social media page information.

We searched the available websites of these programs for 38 items listed in Supplemental Table. Items fell into seven general categories: general program information; application process; research; facility information; current resident information; lifestyle; and social media use. These 38 items were included in our study based on our literature review of previous analyses of residency and fellowship websites in other specialties.^5^–^13^ As descriptive studies, they analyzed a heterogenous list of variables on the websites of interest. The 38 items included in our study are largely based on this literature search.

Understandably, some factors are more important to applicants than others, such as patient volume, curriculum, faculty, research, and simulation training centers.^14^ Although the importance of these factors varies from applicant to applicant, the majority of the variables applicants deem as necessary or desirable information were included in our list of 38 items, in addition to many more items of potential interest we included based on our literature review.^15^ We also added a few additional items to make our study more comprehensive based on items we believe could be lacking from other studies in our literature review, such as social media, resident pictures, and resident hobbies. We also tested the websites for functionality by determining whether the link provided on FREIDA led directly to the residency homepage or required multiple clicks to get to the homepage. The data were collected by three authors (JW, AR, SS) from April 15–May 15, 2020.

As the data contained in residency websites can be subjective, we created a standardized process to evaluate the websites, similar to the previous studies in other specialties.^5^–^9^,^12^ First, we only searched for the presence or absence of items, with no attempt made to grade the quality or accuracy of the content. Second, we excluded any information that was not directly listed on the residency website, such as links to external materials, which may contain general, non-specific information for the program of interest. Lastly, we piloted our search criteria for five programs to resolve ambiguity through independent review by four authors. After this instruction, data collectors independently gathered the data from the 251 remaining websites (HW, AR, SS). When these three authors encountered websites or criteria that were unclear, these criteria were marked and reviewed by a fourth author (JP) and classified accordingly.

We performed a descriptive analysis of the data, including means and standard deviations. To calculate the percentage of items present in each subcategory, we added up the total number of items present among all 249 websites in that subcategory. We then divided this number by the denominator, which was calculated by multiplying the number of variables present in a subcategory by the number of websites examined (249).Microsft Excel 2020 version 16 (Microsoft Corp., Redmond, WA) was used for statistical analysis.

## RESULTS

Of 256 EM residency programs included in our study, seven programs did not have websites on FREIDA and were not accessible by Google search. Of the 249 websites evaluated, 110 websites (44%) provided a direct link from FREIDA to the residency homepage, while 107 (43%) programs required multiple clicks to get to the residency homepage. Thirty-two programs (13%) did not have a link on FREIDA.

**Figure 1:**
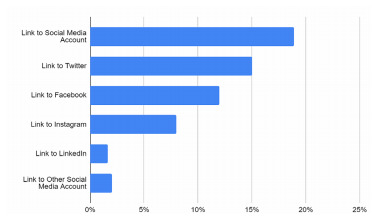
Social media presence of emergency medicine residency programs.

**Figure 2:**
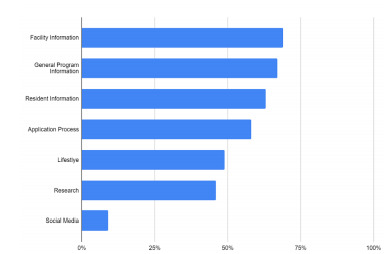
Content available on emergency medicine residency websites.

On average, websites contained 20 of 38 items (53%) with a standard deviation of 6.35. Only 16 programs (6%) contained at least three-quarters of the items of interest. One program contained 37 of 38 items, and no program contained all items. The items that were least commonly available on websites included information on housing and neighborhoods (17%) and social media links (19%) (Table). Within the social media category, the most common forms of social media were Twitter (15%), Facebook (12%), and Instagram (8%) (Figure 1). The items most commonly provided by websites included program description (98%), blocks and rotations (91%), faculty listing (88%), and description for each year of residency (87%). The percentage of each general category is included in Figure 2.

## DISCUSSION

As prospective applicants evaluate EM programs, careful planning and research is essential. The internet is easily accessible, and multiple studies have shown the importance of websites in recruitment.^9^,^12^,^16^,^2^ Our study suggests databases such as FREIDA are useful tools to navigate residency options, with 87% of EM programs providing links to their sites. However, over half of these links required multiple steps, suggesting even this resource could be improved. Our results also demonstrate that many websites are lacking information that is potentially valuable to residency applicants, with an average site missing nearly half of the information we evaluated. We believe enhancing website content could improve the application process for all parties.

The “People” section on websites provided widely varying amounts of information. Despite previous analyses demonstrating that this is the most popular content on EM residency websites,^17^ this information was present on only 63% of EM sites. Similarly, resident biographies and a description of resident hobbies and/or interests were included on fewer than half of EM residency websites. Because an applicant is unlikely to meet all current residents during an interview, resident information on program websites could be the only exposure of such applicants to the unique personalities and backgrounds of residents in the program. Emergency medicine residency programs may benefit from improving these areas of their websites, while being cautious to protect the personal information of their residents.

The presence of social media on residency websites was also limited. Despite the rise of social media for recreational and professional purposes, only 19% of programs contained links to a form of social media for their EM program. In a study done involving nearly 1000 medical students, 68% of students reported using social media to learn about residency programs and 10% reported that the information found in the social media pages influenced their decisions on where to apply.^18^ Similarly, a survey of 142 prospective EM residents led to the recommendation that programs should highlight social activities to improve resident recruitment,^2^ and social media is an efficient way to display social activities. Professional social media integration with EM residency websites appears currently underutilized.

Additionally, lifestyle factors including salary, vacation, meal allowance, and housing costs were often not found on websites. For example, salary information was listed on 63% of websites, vacation time was listed on 57% of websites, and housing and neighborhood information was listed on 17% of websites. Not only are these items important factors to consider when choosing a residency program, but they can be difficult to ask about during an interview, as applicants may worry that asking questions about compensation and benefits give the wrong impression to the program leadership and residents. These topics are discussed during interviews, but these details can be forgotten. Accessible information on lifestyle, which is currently lacking among many EM residency websites, could eliminate this source of potential inquiry or recall concern for applicants.

As the number of residency programs in EM continues to grow,^19^ it has become increasingly difficult for applicants to choose where to apply for visiting clerkships or residency interviews. In 2020. US medical school graduates applying for EM residency applied to an average of 58 programs^1^ despite data from the Association of American Medical Colleges demonstrating diminishing returns for applicants applying to more than 32–39 programs.^20^ While the increased number of applications may be due to the increasingly competitive nature of EM,^21^,^22^ future studies should aim to determine whether lack of online information affects the number of applications. The residency application and interview process is costly for applicants and programs. Providing applicants with more information to guide decisions regarding which programs to apply to and interview at stands to benefit both parties, especially if it results in a better matching of applicants likely to fit a particular program.

The 2020–21 residency application cycle posed a new challenge for applicants and programs. The lack of availability of visiting rotations and in-person interviews contributed to increased uncertainty among applicants, and most were unable to evaluate programs in person. In-person interviews served not only for programs to interview candidates, but for candidates to evaluate programs. Therefore, more than ever, a robust source of information available to applicants on a residency website serves to benefit both applicant and program alike.

## LIMITATIONS

Limitations of our study include the subjective nature of analyzing residency program websites. However, we feel our method of data collection was standardized sufficiently to control for ambiguity. Another limitation was the lack of established standardized criteria for evaluating websites. We based our list of 38 items on our literature search of previous residency website analyses in other specialties, and also relied on papers relating to what EM applicants deem important and the expertise of the authors of our study.^5^–^13^ However, the purpose of our study was not to define the most important items for EM residency applicants, but rather to assess the presence or absence of items on EM residency websites. Inherently, there are items present in our list that may not be very important to some applicants, and items missing which may be important to some applicants.

Future study is needed to provide an updated list of the most important criteria EM applicants could be interested in. The number of programs with “People” sections could be underestimated as many of these programs might have these sections on their social media websites rather than their official residency program websites. Lastly, only including items listed directly on the EM residency website rather than on external links could underestimate the presence of items on websites in our study. However, this was an important factor to determine the accessibility of information and user-friendly status of the websites. Our study does not address accuracy or quality of information contained on websites.

## CONCLUSION

Residency program website quality is important to EM applicants, and our study identifies several areas where programs could focus efforts for website renovation, including improving the integration of social media and providing information on residents and residency lifestyle. The results from our study can be used to improve EM residency websites to the mutual benefit of both applicants and residency programs.

**Population Health Research Capsule**

What do we already know about this issue?

*Residency program websites have been analyzed in other specialties. However, a comprehensive analysis of emergency medicine (EM) residency websites is lacking*.

What was the research question?

*Which components of EM residency websites are most common, and which are least common?*

What was the major finding of the study?

*We identified several areas for website renovation, such as social media integration and residency lifestyle*.

How does this improve population health?

*The results from our study can be used to improve EM residency websites to the mutual benefit of both applicants and residency programs*.

## Supplementary Information



## Figures and Tables

**Table 1:** Presence of items on emergency medicine residency program websites.

General criteria (N=249)	Information found on Emergency Medicine Residency Program websites	% of all Websites
General program information		
	Program description	98%
	Blocks and rotation descriptions	91%
	Faculty listing	88%
	Description for each year of residency	87%
	Message from the program director	69%
	Information for visiting medical students	65%
	Didactic information (A description of didactics or lectures attended)	61%
	Information on tracks and special interests	61%
	Simulation lab information	59%
	Description of each block	48%
	Procedural training nformation	48%
	On-call information	26%
Application process		
	Contact information	78%
	Selection criteria	54%
	Interview dates	53%
	Link to ERAS application	47%
Research		
	Information about research interests and/or active projects	48%
	Information about research requirement	44%
Facility information		
	Description of affiliated hospitals	80%
	Emergency department volume	63%
	Information on the trauma level of the hospitals	63%
Resident information		
	Current residents listed	82%
	Current resident pictures	78%
	Current resident academic history	72%
	Current resident hobbies and/or fun facts	42%
	Current resident biography	40%
Lifestyle		
	Benefits	65%
	Salary	63%
	Vacation and/or sick leave	57%
	Information on surrounding area	48%
	Meal allowance	43%
	Housing and neighborhood information	17%
Social media		
	Link to residency program social media account	19%
	Facebook	12%
	Twitter	15%
	Instagram	8%
	LinkedIn	2%
	Other	2%

*ERAS*, Electronic Residency Application Service.
