# Supplementary material for: Descriptive Analysis of Components of Emergency Medicine Residency Program Websites
Source: West J Emerg Med. 2021 Jul 15;22(4):937–42. doi: 10.5811/westjem.2021.4.50135 (PMC8328176; doi:10.5811/westjem.2021.4.50135)
Supplement: Supplementary file 1 [file 50135_Supplemental_Table.docx]

| General Category | Information Found on EM Residency Program Websites |
| --- | --- |
| General Program Information |  |
|  | Program Description |
|  | Blocks and Rotation Descriptions |
|  | Faculty Listing |
|  | Description for Each Year of Residency |
|  | Message from the Program Director |
|  | Information for Visiting Medical Students |
|  | Didactic Information (A description of didactics, lectures attended, etc.) |
|  | Information on Tracks and Special Interests |
|  | Simulation Lab Information |
|  | Description of Each Block |
|  | Procedural Training Information |
|  | On-call Information |
| Application Process |  |
|  | Contact Information |
|  | Selection Criteria |
|  | Interview Dates |
|  | Link to ERAS Application |
| Research |  |
|  | Information About Research Interests and/or Active Projects |
|  | Information About Research requirement |
| Facility Information |  |
|  | Description of Affiliated Hospitals |
|  | Emergency Department Volume |
|  | Information on the Trauma Level of the Hospitals |
| Resident information |  |
|  | Current Residents Listed |
|  | Current Resident Pictures |
|  | Current Resident Academic History |
|  | Current Resident Hobbies and/or Fun Facts |
|  | Current Resident Biography |
| Lifestyle |  |
|  | Benefits |
|  | Salary |
|  | Vacation and/or Sick Leave |
|  | Information on Surrounding Area |
|  | Meal Allowance |
|  | Housing and Neighborhood Information |
| Social Media |  |
|  | Link to Residency Program Social Media Account |
|  | Facebook |
|  | Twitter |
|  | Instagram |
|  | LinkedIn |
|  | Other |

**Supplemental Table 1.** Items our study evaluated on websites of EM residency programs.
